# ActivePapers: a platform for publishing and archiving computer-aided research

**DOI:** 10.12688/f1000research.5773.3

**Published:** 2015-07-14

**Authors:** Konrad Hinsen

**Affiliations:** 1 Centre de Biophysique Moléculaire (UPR4301 CNRS), Rue Charles Sadron, Orléans, 45071, France; 2 Synchrotron SOLEIL, Division Expériences, St Aubin, Gif sur Yvette, 91192, France

**Keywords:** Computational models, publishing, software tools, scientific record

## Abstract

The lack of replicability and reproducibility of scientific studies based on computational methods has lead to serious mistakes in published scientific findings, some of which have been discovered and publicized recently. Many strategies are currently pursued to improve the situation. This article reports the first conclusions from the ActivePapers project, whose goal is the development and application of a computational platform that allows the publication of computational research in a form that enables installation-free deployment, encourages reuse, and permits the full integration of datasets and software into the scientific record. The main finding is that these goals can be achieved with existing technology, but that there is no straightforward way to adapt legacy software to such a framework.

## Introduction

In the course of a few decades, computers have become essential tools in scientific research and have profoundly changed the way scientists work with data and with theoretical models. Until now, these changes have had little impact on the scientific record, which still consists mainly of narratives published in articles that are limited in size and type of contents, and linked to each other through citations. Some particularly data-intensive fields of research also have their own digital repositories. An early example is the Protein Data Bank ^1^, which publishes and archives structures of biological macromolecules. Some scientific institutions also propose digital repositories for their members, e.g. as part of the Dataverse community ^2^. However, for most domains of research and most scientists, no suitable repositories have existed until very recently, and most datasets are still neither published nor archived.

While the technology used for publishing and archiving the scientific record has shifted from the printing press and libraries to PDF files and Web servers, the kind of information that is being stored has hardly changed. Some scientific journals offer the possibility of submitting “supplementary material” with articles, as a way to circumvent the habitual length restrictions, and for providing unprintable information such as videos. In principle, the data underlying an article can be published as supplementary material as well but this remains an exception and is in fact of little practical interest. The reasons are the various restrictions on file formats and file sizes imposed arbitrarily by different scientific journals, but also the often difficult access to these electronic resources, which usually requires a careful study of each journal’s Web site. Only the recent advent of digital repositories accepting any type of scientific data ^3– 5^ and peer-to-peer networks ^6^ for scientific data has finally made the publication of scientific data accessible to any scientist willing to do so, all the more since these repositories generally do not charge any fees for publication.

The increasing number of mistakes found in published scientific findings based on non-trivial computations ^7, 8^ has made evident the necessity of making computational science more transparent by publishing software and datasets along with any descriptions of the results obtained from them. The fundamental problem introduced by computing is an enormous increase in the complexity of both theoretical models and computational methods, and a similar increase in the size of experimental datasets. These models, methods, and datasets can no longer be handled without a computer, and must therefore be published in computer-readable formats. At the same time, this material must be presented in form that facilitates human understanding as much as possible. The tacit convention in science is that published results are assumed correct unless there is clear evidence suggesting a mistake. As C.A.R. Hoare famously said ^9^, “There are two ways of constructing a software design: One way is to make it so simple that there are obviously no deficiencies, and the other way is to make it so complicated that there are no obvious deficiencies.” This observation applies equally to computational science, where the vast majority of published results have no obvious deficiencies, but are obtained using software that is much too complicated for anyone, authors and readers alike, to be certain about its correctness.

While publishing software and other electronic datasets is now technically possible, and initiatives have been started to create incentives for scientists to invest the additional effort required for making such material available ^10^, much more work remains to be done to ensure that published software and datasets can actually be understood, verified, and reused by other scientists. This is particularly important because computational methods are becoming an essential aspect of all scientific research, including experimental and theoretical work in which computations are not the main focus of activity. It is therefore more appropriate to discuss these issues in the context of “computer-aided research” rather than the more narrow specialty called computational science.

Most current efforts in this direction (see e.g. Ref. 11– 13) start from the *status quo* of computation in science and propose small-step improvements in order to facilitate adoption by the scientific community. The work presented in this article takes the opposite approach of starting from the requirements of the scientific record and exploring how software and electronic datasets need to be prepared in order to become useful parts of this record. Both approaches are complementary: while ease of adoption is important for rapid improvement, it is also important to have a clear idea of the goal that should ultimately be reached, in order to avoid getting stuck in technological dead-ends.

This article presents the lessons learned from developing a new computational framework, called ActivePapers ^14^, and from using it for several research projects in the field of biomolecular simulation. The first section discusses the conditions that electronic datasets, including software, must fulfill in order to be fully integrated into the scientific record. Next, the concept of an ActivePaper as a collection of electronic datasets with references and provenance information is introduced. A brief overview of tools developed by the Reproducible Research and Open Science communities explores similarities to and differences from the definition of an ActivePaper. Two concrete realizations of the ActivePapers idea are presented, and experience with their use in several research projects is reported.

The main contribution made by this work are the following observations and insights:

- The preservation of scientific software is important for maintaining the scientific record.
- Ease of software deployment is important for verifying the replicability of computer-aided research.
- Preservation and ease of deployment are related problems that can be solved together.
- The required technology exists today, but is not popular in the scientific community.
- Effective use of such approaches requires a separation of “computational tools” and “computational models and methods”. Models and methods can be archived in the scientific record, whereas tools can be verified to implement these models and methods and need not necessarily be preserved.
- Preservation and ease of deployment are very difficult to ensure for existing scientific software. They must be taken into account from the design stage.

## The scientific record

The term “scientific record” refers to the totality of published scientific findings in history. It started to become organized in 1665 with the creation of the first scientific journal, the Philosophical Transactions of the Royal Society. A scientific journal publishes articles, which are narratives explaining the motivation for a specific study, the methods being applied, the observations made, and the conclusions drawn. The exact observations are provided in the form of tables and figures.

The scientific record plays two main roles in the process of scientific research. The first role is to ensure the transparency and reproducibility of scientific results, which is an important measure of protection against both mistakes and fraud. The second role is to permit future research to build on the results of the past, to “see further by standing on the shoulders of giants”, as Isaac Newton expressed it.

One of the main characteristics of the scientific record is its permanence. Once an article is published in a journal, a permanent reference is attached to it, and the publisher accepts the moral obligation to make the information accessible through this reference for as long as possible. Traditionally, this reference is a citation taking a more or less standardized format. With the transition to electronic publishing, the role of the permanent reference is fulfilled by a Digital Object Identifier (DOI) ^15^, which is defined by the international standard ISO 26324:2012. For published articles, permanence also applies to the contents: once published, they cannot change. Mistakes detected after publishing can be corrected only by publishing a separate short article called “erratum”. More recently, the idea of versioning has been applied by some publishers, e.g. F1000Research: a DOI refers permanently to a specific version of an article, but multiple versions of this article remain accessible permanently in order to document the evolution of the publication.

The permanence of the scientific record applies only to the preservation of the original expression of each information item, but not to its semantics. A published article can become unintelligible because of changes in terminology and in the scientists’ education. A modern physicist would not recognize the theory of classical mechanics in Newton’s “Philosophiæ Naturalis Principia Mathematica” ^16^ without prior training in Latin and in the history of science. Scientific work that has been recognized as important is reformulated and summarized in the course of time in the form of review articles, monographs, and textbooks for education. For this reason, the slow decline in intelligibility of the original publications is in general not a problem.

With the widespread adoption of computer-aided research methods, the size and complexity of scientific data has exploded, making a publication in the form of tables and figures both impracticable and useless. Digital repositories have therefore become an important second pillar in several domains of research, next to the traditional journals. These repositories store electronic datasets submitted by researchers and attach metadata such as author names, date of submission, file formats, or citations of related journal articles. These datasets are made available to the scientific community, usually free of charge, for use in other studies, including the application of data mining techniques in meta-analysis studies.

Permanent references to electronic datasets take the same form as for electronically published journal articles, i.e. a DOI, and the repositories have the same moral obligation as journal publishers to keep the datasets accessible as long as possible. Concerning the permanence of the contents, each repository applies its own policy. Zenodo ^5^, for example, does not allow any modification of published datasets, but allows a dataset to be marked as obsolete with a reference to its successor. The Protein Data Bank (PDB) ^1^ updates its electronic records to correct mistakes in the representation of the data and to adapt the data to changes in the PDB’s data model. However, changes to the scientific contents are not permitted and require the submission of a new entry, which like in the case of Zenodo can be labeled as making a prior entry obsolete.

The most important difference between traditional articles and electronic datasets from the point of view of the scientific record is the semantic context required for the interpretation of the stored information. For articles, the semantic context consists of a human language and the jargon and conventions of a specific scientific discipline, which change very slowly, on a timescale of centuries. For electronic datasets, the semantic context is ultimately defined by the computers and programs that read and write the data, which can change, intentionally or by accident, in a few seconds. The longevity of electronic datasets therefore relies on a careful documentation of the data models and data formats being used, on careful testing of software to ensure that this documentation is respected, and on proper curation of submissions by the repository managers to ensure adherence to these formats.

Science librarians have developed tools (e.g. 17) and documented best practices for the preservation of digital artifacts. However, applying these methods implies an additional processing step for converting the files produced during research to formats suitable for archiving. This step represents an additional effort and increases the risk of introducing mistakes. Many scientists prefer to use the latest generation of uncurated digital repositories ^3– 5^, which accept any computer file with no requirements concerning data formats or their documentation. It is to be expected that much of the information in these repositories will remain accessible for a long-time but quickly become unusable. Ideally, scientists should therefore use archivable data formats in all phases of computer-aided research. This is one of the goals of the ActivePapers project.

### The challenge of software preservation

Among the electronic datasets used in computer-aided research, software poses the most difficult challenges for preservation because of its semantic complexity. What exactly a piece of scientific software does is defined by (1) its source code, (2) the programming language it is written in, and (3) all its dependencies: libraries, other programs called as subprocesses, etc. Since many of today’s popular programming languages are not defined with precise semantics (see e.g. Ref. 18 for the languages C and C++), (1) must in practice be replaced by the specific compiler or interpreter being used. Moreover, compilers, interpreters, and dependencies are in constant evolution. Preserving a piece of scientific software with its exact semantics would thus require preserving its complete computational environment down to the hardware.

For this reason, many practitioners consider the idea of preserving scientific software for many years unrealistic, and some even argue that it is unnecessary because computational methods change so rapidly that their long-term preservation is of no interest. The latter argument is manifestly not valid in general. As an example, the DSSP method for defining secondary-structure elements in proteins ^19^ was published more than thirty years ago and is still widely used today. The example of DSSP is also interesting in that the most widely used software implementing DSSP today ^20^ does not in fact implement the exact method published in the original paper. The differences are not documented anywhere at this time, and scientific papers using the modern software systematically cite the original paper without further comment. It can thus be assumed that most DSSP users are not aware of the fact that they are using a modified method. If the original method had been published in executable form and preserved until today, such discrepancies could have been avoided.

Software preservation has only recently become a topic of research in its own right. One focus is the definition of the features of software that can or should be preserved ^21^. Various technologies for preserving software can then be evaluated with reference to this feature list. Other projects (e.g. 22) start by trying to preserve the software behind a given scientific study in order to explore the issues that need to be addressed. The ActivePapers project pursues the inverse approach: rather than working on the preservation of today’s scientific software, it explores how tomorrow’s scientific software could be written in a way that facilitates preservation.

It is interesting to note that software preservation is strongly related to software distribution and deployment, which are generally recognized as important and difficult technical problems. Deployment means making a specific piece of software available for use in a given computational environment, whereas distribution addresses the question of packaging software in a way that facilitates deployment. Deployment and preservation are difficult for the same reason: the enormous diversity and fast rate of change of computational environments. It can thus be expected that progress in software preservation will also lead to simpler deployment, a change that computational scientists would certainly welcome.

### Replication, reproduction, and reuse

One reason for maintaining the scientific record is to support the fundamental error-correction mechanism of the science, which consists of allowing anyone to verify prior scientific studies by trying to reproduce them. Reproducibility is indeed one of the cornerstones of the scientific methods. It is what distinguishes a scientific study from an anecdotal report.

The reproducibility of computational approaches in science has received significant attention recently as a consequence of several highly visible computational studied that turned out to be mistaken ^7, 8^, causing a credibility crisis in computational science. The most basic requirement for building confidence in computational results is total transparency: the publication of all datasets and software used during a scientific study. This is the main goal of the Reproducible Research movement, which has been gaining traction over the last years ^23– 25^. The focus of these activities is transparency but not preservation, which most members of the Reproducible Research community consider desirable but unrealistic. The tacit assumption is that reproduction attempts should be done rapidly while computational environments similar to those in the original work are still easily available. Nevertheless, the developments in Reproducible Research have shaped a more precise picture of the role of software and computation in scientific research, which is important when considering how to integrate this information into the permanent scientific record.

An important distinction in this respect is the one between *replication* and *reproduction*. Replication refers to an attempt to repeat a computational protocol under conditions that are as similar as possible to the original setting. It is essentially a quality-control measure: if scientist B can replicate scientist A’s computation, this shows that A has provided a sufficiently precise, complete, and correct record of the original work. Replicability is far from trivial because a precise and complete description of a computational study is a dataset that is both large and complex. Moreover, it is often difficult to obtain in today’s computing environments. Reproduction refers to an attempt to repeat a computational study following the general principles of the original work but using different tools. Reproducibility plays the same role as in other branches of science: it establishes which aspects of a computational protocol are important for reaching specific conclusions, and identifies unintended dependencies of results on technical details of the procedure.

Replicability and reproducibility are not completely independent. For all but the simplest computational methods, reproducibility requires replicability. If a reproduction attempt leads to significantly different results, the cause of the differences must be explored. This is in practice only possible if the original results can at least be replicated. Otherwise, the most probable explanation for the difference is that the original study was insufficiently documented.

Since replicability is a purely technical aspect, it would ideally be verified upon submission of a computational study, as part of the reviewing process. In practice this does not happen yet, because the difficulty of software deployment puts an important cost on replication attempts. Assuming that these problems will be overcome either by reducing the cost or by accepting to pay it, replication will become a one-time process and there is no reason to perform replication attempts on published scientific studies. The preservation of electronic datasets, including software, in the scientific record therefore has reproducibility as its main goal. The second important goal is to permit *reuse* of both data and computational methods. This corresponds to the second role of the scientific record, which is to allow future research to build on work from the past.

Both reproduction and reuse add an important requirement to the technical criterion of replicability: the original computational protocol must be understandable by the scientist who sets out to explore the failure of a reproduction attempt, or to apply the protocol to a different problem. Experience has shown that understanding a computational protocol is based both on inspection of the source code and on experimentation, i.e. observation of the programs’ behavior for various input data. Any software preserved in the scientific record must therefore allow both inspection and execution.

Unfortunately, today’s scientific software tends to be even more complex than the methods it implements because it also needs to take into account complex technical issues such as performance and resource management. This conflation of scientific and technical aspects makes the software both more difficult to inspect and more difficult to preserve because of platform-specific optimizations. Ideally, these two aspects should be separated. The scientific contents, i.e. models and methods, should be preserved in the scientific record as specifications written with readability by humans in mind. Software tools should then read these specifications and perform optimized computations based on them. The state of the art in computational technology does not permit such a separation at this time, but I have outlined possible solutions to this problem in Ref. 26.

## The ActivePapers project

The goal of the ActivePapers research and development project is to design a platform for publishing and archiving computer-aided research whose first priority is to ensure the utility of electronic artifacts as items in the scientific record. Such a platform should ideally meet all of the following requirements:

1. A published electronic dataset, in the following called an ActivePaper, should be allowed to contain a combination of data, code, and narrative related to a research project.
2. An ActivePaper can reuse data items from previously published ActivePapers via references. These references can also be used for attributing credit to everyone involved in producing the information that is reused.
3. The representation of executable code inside an ActivePaper should be well-defined, stable, and sufficiently simple to allow implementation on future computing systems with minimal effort. The execution of any piece of code from an ActivePaper should always produce exactly the same results at the bit level.
4. Any code stored in an ActivePaper should be safe to execute, i.e. it should not be able to cause any harm to the computing environment it is executed on.
5. An ActivePaper should contain metadata for provenance tracking and reproducibility.

In view of the planned use of this platform for biomolecular simulations, additional requirements follow from the needs of high-performance computing on big datasets:

6. An ActivePaper should support large datasets by ensuring compact storage and high-performance data access.
7. No data conversion should be required to prepare an ActivePaper for long-term preservation, or to reuse data from an archived ActivePaper.

While the first requirement states that an ActivePaper should be able to combine data, code, and narrative in a single package, this does not preclude a more fine-grained organization of the results of a scientific study in the form of multiple ActivePapers. In particular, it is possible to publish individually raw data sets and software libraries, the two research outputs that are the most suitable for reuse.

The utility of bit-level replicability is controversial in the scientific communities that make heavy use of numerical computations. It is difficult to achieve in practice today, because replicability was not a priority in the design of today’s computing technology. The most widely known difficulty is compiler optimizations of floating-point operations that tend to introduce uncontrollable approximations into a computation. However, a change in any external dependency (library, development tool, ...) can modify the output of a program as a result of mistakes or well-intentioned improvements. Bit-level replicability is also not strictly necessary for any given simulation, whose result is inaccurate anyway. In principle, it is sufficient that the results from different executions agree to the precision at which the result is considered accurate. However, any criterion for defining if results are “close enough” for replication depends on the nature of the problem being solved. At the level of a computational platform, the only possible guarantee is bit-level replicability. Moreover, bit-level replicability is indispensable for software testing ^27^.

The fourth requirement, security of execution, could be satisfied outside of the ActivePapers platform, by embedding the platform’s run-time support software into a suitable sandboxing mechanism. However, this puts the burden and risks of implementing security on the user of the platform.

It is important to note that it is *not* required that all software used for a computational study be stored in ActivePapers. On the contrary, it is to be expected that important software tools remain forever outside of the ActivePaper universe and work on ActivePapers as data. This includes everything requiring user interaction, from authoring tools to data visualization programs, and also highly machine-specific software such as batch execution managers. It is also possible to write external code accelerators that take code from an ActivePaper and execute it after optimization and/or parallelization, guaranteeing identical results. While the current state of the art does not provide techniques for making such code accelerators both general and efficient, it is possible and even straightforward to write problem-specific code accelerators, which are simply efficient reimplementations (in a language like Fortran or C) of algorithms stored in an ActivePaper, with the equivalence of the results verified by extensive tests.

The requirement of metadata for provenance tracking and replicability implies that an ActivePaper must contain sufficient information to reconstruct the full dependency graph of all datasets. Each dataset that is the result of a computation must have a reference to the program that generated it plus a reference to all other datasets that this program has used as input.

The notion of an ActivePaper bears many resemblances with the concept of a Research Object that has recently been proposed ^28^. In fact, an ActivePaper can be considered a realization of the Research Object idea with particular emphasis on the computational aspects of research.

### Evaluation of existing technology

Some of the requirements listed above overlap with requirements from other domains, suggesting a potential for reuse of existing technology. A brief survey is given in the following.

### Tools for reproducible research

The Reproducible Research movement addresses many of the needs of publishing computational science, with the notable exception of long-term preservation. It is therefore interesting to consider the tools that have been developed for making computational research more reproducible.

The *computational notebook* approach ^13^, pioneered by Mathematica ^29^ and recently popularized by the Jupyter project (formerly known as the IPython notebook) ^30^, builds on earlier developments in literate programming ^31^, which have also been applied to computational science directly ^32, 33^. It aims to integrate computational methods expressed as working code with input/output data and the scientific narrative. It permits a seamless transition from interactive exploratory work to a documented computational method that can be shared and published. Compared to traditional scripts, computational notebooks represent an important advance in improving reproducibility through improving human understanding. However, none of the existing notebook implementations fulfill any of the requirements listed above for an ActivePaper. Like a script, a notebook depends on the computational environment in which it was generated. This environment is neither preserved nor even documented in the notebook. After a few years, a notebook still provides a human-readable and rather detailed description of the method, but re-running it is likely to be difficult or impossible. Moreover, the notebook approach does not take into account datasets, unless they are small enough to be included as literal data into the notebook itself. All other data is accessed by usually non-permanent references such as filenames or Universal Resource Locators (URLs).

Similar remarks apply to workflow management systems such as Kepler ^34^, Taverna ^35^, or VisTrails ^36^. In fact, workflows, scripts, and notebooks all refer to the same basic concept: the outer algorithmic layer that defines a specific computational study in terms of more generic components. The differences lie in the user interface and in the kind of components that can be used (libraries, executables, Web services, etc.). Some workflow managers can archive these components partially, and also some kinds of datasets, but such support is neither complete nor exhaustive. The one aspect in which workflow managers improve on notebooks is the fifth ActivePapers requirement, i.e. provenance tracking.

Attempts to record a computational environment in a form that can be restored on a different computer are still rare. Ref. 37 describes how this goal can be achieved for programs in the R language, extending that language’s package and repository mechanisms by package libraries, package cohorts, and validated repositories. There are many similarities with the ActivePapers approach to software preservation and deployment (requirement 3 in the list given above). It is also interesting to note the main difference: the tools switchr and GRANbase described in Ref. 37 start from the R language’s existing mechanisms and extend it with functionality required by the scientific record, whereas ActivePapers starts from existing mechanisms for publishing electronic artifacts and extends them to include software.

The most comprehensive approach to archiving scientific software in an executable form is based on virtual machine technology ^38– 41^. The authors of a computational study produce a virtual machine image that contains their complete computational environment, starting with the operating system, in addition to the problem-specific data and workflows. A published virtual machine can be used safely by others for replication, fulfilling requirement 4 and partially requirement 3 of the list given above. However, this approach also has some significant disadvantages. The resulting archives are in general too big to be deposited in today’s general-purpose digital repositories. Moreover, it is not possible to refer to or reuse individual pieces of software or data inside a virtual machine image, nor is it straightforward in general to analyze the software or data except by the tools explicitly provided by the authors of the virtual machine. Virtual machine technology effectively creates a technical barrier to fulfilling requirement 2. The longevity of archived virtual machine images is uncertain, making requirement 3 impossible to fulfill in the long run. Executing such an image requires complex and sophisticated software, which for the moment is produced and maintained by non-scientific organizations for reasons completely unrelated to science. Once technological progress makes these efforts obsolete, it must be expected that computations archived as virtual machine images will become unusable. In summary, virtual machines are a good solution to facilitate deployment for replication attempts, but are not a suitable data format for preservation.

Finally, the Exec&Share server ^42^ and Elsevier’s Collage Authoring System ^43^ propose the creation of “companion sites” to journal articles, which contain both software and datasets. A Web interface permits users to run the software on the authors’ input data or on their own data sets. In terms of the ActivePapers requirement list, a companion site fulfills points 1, 3, and 4. The main advantage of this approach is the ease of deployment for replication or reuse, which requires no software installation. It is the server administrators’ responsibility to maintain the computational environment for the deposited software. For this reason, there are severe restrictions on the kind of software that can be deposited, and similar restrictions apply to the format and size of the deposited datasets. Long-term preservation is technically possible, but at this time none of the operators of these services has made any promise of long-term engagement for keeping the contents available in a functional state.

### Bytecode platforms

The term *bytecode* (sometimes also called *p-code*) describes an instruction set for a hypothetical processor. A compiler that generates bytecode instead of machine code for a hardware processor typically produces smaller compiled code which moreover does not depend on a specific hardware platform. However, since bytecode cannot be executed directly by a physical processor, it requires an additional processing step, either interpretation or a second compilation phase, which tends to reduce overall performance. The principle of compilation to bytecode has been used for almost 50 years, initially with the goal of generating compact and portable code, the loss of performance being accepted as a trade-off. More recently, just-in-time (JIT) compilation techniques have significantly reduced the performance issues related to bytecode to the point that there is often no significant performance difference to programs compiled to native machine code.

The use of bytecode is often an implementation detail of a specific programming language, with no directly visible consequence to its users. But bytecode can also be the starting point for the definition of a *bytecode platform*, such as the Java Virtual Machine (JVM) ^44^ or the Common Language Infrastructure (CLI) ^45^. A bytecode platform is a specification that combines a bytecode instruction set with a runtime library that provides access to system resources (memory, threads, files, network, etc.). A program written for a bytecode platform that relies only on this standard library for interacting with its environment is fully portable in the sense that it can run unmodified on any computer for which an implementation of this platform exists. In addition to portability, bytecode platforms also offer interesting security features. The runtime system processing the bytecode can offer much more fine-grained access control to system resources than today’s operating systems, making bytecode platforms a good choice wherever there is a risk of malicious code distribution (viruses etc.). In summary, a well-designed bytecode platform implements points 3 and 4 of the ActivePapers requirements list.

The notion of a platform as a precise and complete specification of the computational environment that a piece of software relies on is of particular importance for software preservation. In fact, software preservation requires preservation of the files containing the software plus preservation of the platform the software was created for. Most of the well-known difficulties with software distribution, deployment, and preservation have their roots in the lack of well-defined computational platforms, as does the frequent non-replicability in computer-aided research. As an example, the platform for software distributed as source code consists of the language specification, if it exists, or of the compiler or interpreter otherwise, but also of the specific versions of specific libraries used by the program. Frequently the operating system is also part of the platform specification, because the program itself or one of its dependencies relies directly on operating-system services to access system resources. With such platform specifications being complex, informal, and numerous, most software authors target not one platform but a family of related platforms (e.g. “Unix-like systems with a C99 compiler”), and leave the work of getting all the pieces work together to packaging experts, systems administrators, and end users.

Because of the importance of stable platforms for both replicability and software preservation, it is useful to look in some detail at the track record of today’s most successful bytecode platform, the JVM. It was originally defined as a support for running software written in the Java language, but by now the JVM hosts a variety of languages and ensures a high level of interoperability between them. The goal of the JVM developers was to enable the distribution of executable code via the Web, which users could run in their browsers without any prior installation or configuration. The main challenge resulting from this goal was the distribution and deployment problem: executable code deposited on a Web server had to “just work” on any computer equipped with a JVM implementation and a Web browser. This goal has overall been reached successfully, and with remarkable stability: JVM bytecode produced in 1995 can still be run without modification.

The JVM’s approach to distribution and deployment is that both libraries and application programs are packaged as *JAR files*, which can contain JVM bytecode files but also additional resources such as data files read by the software ^46^. Executing a JVM application requires specifying explicitly all the JAR files it consists of. In practice, most application programs are distributed as a single JAR file that contains all dependencies other than the JVM and its standard library. A JVM application is thus a plain file that can be distributed and archived just like any other file. In contrast, the traditional approach to managing software is to consider libraries and application programs as extensions to the operating system that are “installed” one by one, with each new package being allowed to rely on previously installed packages. Many of the difficulties encountered in deployment have their origin in this requirement of every software package being part of a single installation, which doesn’t leave enough flexibility for solving conflicts between the requirements of different software packages. The JVM approach neither prevents nor resolves such conflicts, but shifts the responsibility for dealing with them from the systems administrators and end users to the person packaging an application program for distribution.

It is instructive to look in more detail at the two aspects in which the JVM platform failed to attain universal portability: (1) interfacing to certain operating-system services, such as user interface layers or concurrency management, and (2) floating-point computations.

The limited portability of access to certain aspects of the computational environment is due to a combination of high diversity among different operating systems and the difficulty of making compromises. For graphical user interfaces, any compromise leads to a bad user experience, and thus a lower acceptance of the software. For concurrency, compromises lead to bad performance, with similar consequences. Another problem specific to concurrency is the difficulty of providing an exact specification of a system’s behavior in all imaginable circumstances.

Fortunately, neither of these is an issue for pure computations, i.e. software that transforms input data into output data but does not interact with its environment in any other way. This observation is important because the scientific aspects of software are always pure computations. The definition of “scientific aspect” here is “everything that has an influence on the results of computations”. Any result is represented in the computer as finite sequence of bits, which can be considered the value of a mathematical function of all the input data, which are finite bit sequences as well. This implies that all results can be obtained from pure computation. This fact is not obvious because in most of today’s scientific software, the scientific aspects are closely entangled with technical aspects such as user interaction or resource management. As an example, a typical visualization tool entangles the mapping from input data to graphics primitives, which is a pure computation, with the code for displaying these primitives on a computer screen and letting the user interact with the display. A carefully designed software architecture can separate the scientific contents from the user interface aspects and ensure replicability and preservation for the scientific aspects.

The limited portability of floating-point operations on the JVM is due to a deliberate decision to give up their precise initial specification in favor of a less rigid one that permits a more efficient implementation on today’s most widely used hardware architecture, Intel’s x86 series of processors. It is still possible to choose the original precise floating-point semantics, but few programmers make that choice. This illustrates one of the main reasons for the lack of rigorous specifications of computational platforms: the desire to leave implementers as much room as possible for optimizing performance on the currently relevant hardware platforms. In the case of floating-point operations, which are particularly important in scientific computing, the widespread but wrong belief that they are inherently imprecise has contributed by making most computational scientists accept the *status quo* without discussion. This attitude is slowly changing because of the problems becoming ever more visible in several domains of scientific research (see e.g. 47– 49). Reproducibility in floating-point arithmetic has become an active topic in computer science research (see e.g. 50– 52).

The second popular bytecode platform, the ECMA standard CLI ^45^, can be considered a more modern implementation of the basic JVM idea, with a change in priorities. The CLI platform was designed by Microsoft and implemented as the .NET framework for Windows. The .NET framework, which also contains libraries that are not part of the CLI platform, remains the dominant CLI implementation, to the point that much software described as CLI-compliant has in fact never been tested with any other implementation. Portability of .NET/CLI applications is therefore more limited than with the JVM. Software developers use .NET because of its security features, ease of deployment, and safe interoperability between multiple programming languages. It is in particular the last point where the CLI platform has clear advantages over the JVM, which was originally designed for a single language, Java.

A third bytecode platform, Google’s Portable Native Client (PNaCl) system ^53^, is so recent that it cannot be evaluated based on practical experience. Its design goal is to permit the execution of application software inside a Web browser, which resembles the original goal of the JVM. However, PNaCl uses a more low-level code representation than the JVM or CLI, which is defined by the LLVM project ^54^, and a less precisely defined runtime environment, in order to facilitate the adaptation of software written in traditional programming languages such as C and C++.

### The ActivePapers JVM edition

The original ActivePapers architecture ^55^, which was subsequently implemented in the “ActivePapers JVM edition”, was a proof-of-concept design intended to show that it is possible with existing technology to meet all the requirements defined above. The key design and implementation choices were

- An ActivePaper is a file in HDF5 format ^56^. The HDF5 format ensures flexibility, compactness, and high-performance data access (requirements 6 and 7).
- Any data item inside a published ActivePaper can be referenced by the combination of the ActivePaper’s DOI and the HDF5 path to the dataset (requirement 2).
- Executable code is stored as JVM bytecode. Any other code representation, in particular human-readable source code in any language, is admissible if a compiler or interpreter exists in the form of JVM bytecode (requirement 3).
- The JVM security model is used to prevent executable code in an ActivePaper from accessing any data outside of the ActivePapers platform, enforcing security at the platform level (requirement 4).
- HDF5 dataset attributes are used to store metadata, including a dataflow graph that records provenance (requirement 5), but also creation time stamps and a data type indicator distinguishing references and executable code from “plain” datasets.
- Individual programs inside an ActivePaper are labeled as either pure computations (“calclets”), or as data importers (“importlets”). The latter have unrestricted read access to anything, including local files and network resources. They share the write restrictions of pure computations, meaning that they cannot modify anything outside of the ActivePapers universe. Moreover, they are never run automatically, but only on explicit user request.

The original design included a third kind of programs, “viewlets”, intended for visualization or more generally for letting users inspect the results of computations, possibly interactively. This idea was abandoned, at least for the initial implementation, because of the difficulty of achieving efficient yet portable user interaction on the JVM platform.

An implementation of the original ActivePapers platform is available from the ActivePapers Web site ^14^. Its only dependencies are (1) a Java Virtual Machine implementation, (2) the HDF5 library, and (3) JHDF5 ^57^, a Java interface to the HDF5 library. The ActivePapers software provides a command-line interface for creating ActivePapers, inspecting their contents and metadata, and for running the embedded executable code. This is clearly a minimal working environment. For production use by a wide community of computational scientists, many convenience functions would have to be added: a code and data editor, data visualization, data management, etc.

The use of the JVM bytecode platform by itself ensures that most of the requirements listed in the beginning of this section are met. In fact, many of these requirements could be met by using JAR files as ActivePapers, adding just a set of conventions for storing data, metadata, and references inside JAR files. The use of the HDF5 format rather than the JAR format for ActivePapers is motivated by the requirement of efficient handling of large datasets. The JAR format, which is a variant of the ZIP format, is designed to be compact and provide efficient read access to individual files, but it does not provide efficient write access nor efficient random access to parts of files. The HDF5 format is much more general, and in particular allows performance tuning for various data access patterns. The HDF5 library also offers much useful functionality for structuring data access in scientific software. Finally, HDF5 is already widely used in scientific computing. One of the dataset types offered by HDF5 is a one-dimensional array of bytes, which is exactly the data model for files in today’s operating systems. An ActivePaper can therefore store arbitrary file contents in a dataset, a feature which it uses to store JAR files for executable code.

A disadvantage of the HDF5 format is the increased complexity of the ActivePapers platform, which includes HDF5 and JHDF5 as dependencies. It would be preferable to be able to access HDF5 files from pure JVM code, reducing the additional dependency to one more JAR file. It is possible in principle to reimplement HDF5 in Java, and partial implementations already exist, but they are insufficient for practical use at this time.

Two of the requirements for an ideal ActivePapers platform require a more detailed discussion with respect to the choice of the JVM. The representation of executable code was supposed to be simple, in order to facilitate implementation on future computing systems, and all results were supposed to be replicable at the bit level. A modern JVM implementation with a JIT compiler and a complete runtime library is a large and complex piece of software, and its current imprecise floating-point semantics do not allow full bit-level replicability. However, the complexity of today’s JVM implementations is mostly due to performance optimizations. JVM bytecode is rather simple, and a working but inefficient implementation is not difficult to realize. As for bit-level replicability, it could be ensured in two ways: (1) allow only JVM bytecode in which all functions are compiled in strictfp mode, or (2) use a special JVM implementation that always uses the initial strict floating-point semantics.

An important design decision is related to the management of the metadata that tracks dependencies and provenance. The ActivePapers platform creates and updates this metadata automatically during program execution. From the user’s point of view, an ActivePaper is a collection of datasets and programs, of which the latter can be run individually just like traditional executables or scripts. The ActivePapers platform tracks all data accesses from programs and generates the dependency graph from them. When a program is re-run, typically after modification, all the datasets it generated earlier are deleted automatically. Moreover, when a program reads data generated by another program which has been modified since it was last run, the modified program is re-run automatically to ensure coherence of all data. This automatic dependency handling has worked well in practice. It is the inverse of the approach taken by automation tools such as make ^58^, which execute programs according to a manually prepared definition of the dependencies between their results. Note that the difference concerns only *how* the metadata is created. The resulting dependency graph is the same with both approaches, and the choice between them is not a fundamental one, but mainly a user interface design aspect.

The ActivePapers implementation for the JVM provides an automatic replication tool. It analyzes the dependency graph of an ActivePaper and re-runs all computations in the required order. The result is a new ActivePaper file that can be compared to the original using the tool ‘h5diff‘ from the HDF5 distribution. Ideally, the two files should have identical contents except for the time stamps. This has worked very well for all tested examples, even in the presence of floating-point computations. However, all tests were performed on machines with a processor from the x86 family, for lack of access to any significantly different machine. Automatic replication is a promising approach for validating computer-aided research at submission time.

The JVM security model was integrated into ActivePapers with the goal of protecting the user’s computing environment against damage done by erroneous or malicious code. The platform was never used widely enough to generate any experience with malicious code, but the security mechanisms have proven very helpful in detecting errors. A typical situation is the transfer of code written for stand-alone use into an ActivePaper. The original code thus accesses local files and network resources for input and output. These access must be modified to work exclusively on ActivePaper datasets. The JVM access control mechanisms were very helpful in locating these accesses and in ensuring that every single one was taken care of.

While the ActivePapers JVM edition satisfies all the initial requirements, it is very difficult to use in real research projects, because of a critical lack of scientific software compatible with its constraints. All code running inside of the ActivePapers platform must exist as JVM bytecode. All code storing data in an ActivePaper must use the HDF5 library. All code that falls into both categories, which includes in particular the workflow of a specific research project, must exist as JVM bytecode accessing the HDF5 library. There is almost no publicly available code that meets these requirements, due to the lack of popularity of the JVM in scientific computing.

### The ActivePapers Python edition

In order to gain experience with the ActivePapers approach in practice, a second implementation was developed for the Scientific Python ecosystem ^59^. Its dependencies are the Python language ^60^, the HDF5 library, the h5py library ^61^ for interfacing HDF5 to Python, and the NumPy library ^62^ which is a dependency of h5py. For the Python edition of ActivePapers, all executable code must exist in the form of Python scripts, which access the datasets through the h5py library. Python libraries that contain extension modules (which is Python jargon for modules written in a compiled language such as C) cannot be placed inside an ActivePaper, but can be declared as an external dependency. This effectively means that the platform required for using an ActivePaper with such a dependency includes that library in addition to the packages listed above. Adding external dependencies is clearly not desirable, but it provides a short-term workaround to the fundamental problem that most scientific software is not ready for installation-free deployment and long-term preservation.

The Scientific Python ecosystem provides a large choice of libraries that can be used within these constraints, and the Python language is already very popular for scientific computing, making the ActivePapers Python edition a good vehicle for testing the ActivePapers approach on real research projects. On the other hand, the Python edition cannot fulfill requirements 3 and 4 listed above. In particular, the Python language lacks sufficiently strong security mechanisms to implement a useful level of user protection (requirement 4). A more subtle problem is the stability of the platform itself. The Python language has no formal specification and in fact evolves together with its principal implementation. The scientific libraries, in particular NumPy, also evolve rather rapidly, with only moderate efforts to maintain compatibility with older versions. For example, version 1.9 of NumPy removed a compatibility layer for use with older Python code that was written for NumPy’s predecessor Numeric. It is difficult to estimate how much published research code was affected by this change, but it includes everything I have published between 1997 and 2012. The ActivePapers platform records the version of all libraries that were used in the preparation of an ActivePaper, but the long-time usability of these versions is questionable, as in general only the current versions can be expected to work in current computing environments. Requirement 3 is therefore only partially fulfilled.

The ActivePapers Python edition has been used for several research projects in the field of biomolecular simulation, some of which have already been published ^63– 65^. Each publication has one or more ActivePaper files deposited as supplementary material, but all the files are also available in digital repositories with DOIs. Among the published ActivePapers, there are software libraries ^66, 67^, a database of protein structures ^68^, and combinations of datasets and code that document computational studies ^69– 71^. Additional published ActivePapers contain obsolete versions of the pyMosaic library ^72– 74^. These files remain permanently available because other ActivePapers depend on them. They also remain usable for as long as the underlying platform remains compatible.

Many of the published ActivePapers reuse code or data from other published ActivePapers through references. Each reference consists of the DOI of the ActivePaper file that contains the data plus the HDF5 path that identifies the dataset inside the file. The ActivePapers management software automatically downloads ActivePapers that are required for satisfying references. This download mechanism supports Zenodo ^5^ and figshare ^4^ at the moment. It would be desirable to support any DOI-granting digital repository, but unfortunately the DOI infrastructure does not yet provide the required functionality. DOI resolution produces a URL pointing to a human-readable landing page. This page typically contains links to the data files, but its layout is not standardized, making it impossible to find these links automatically and reliably.

The possibility of packaging code and data together has turned out to be very useful. In deciding how to divide the complete information resulting from the research projects into ActivePapers, the major considerations were the following:

- What makes a useful package for reuse?
- Which information must be consulted together for understanding the computational methods?
- Which items are required to back up the claims of a journal publication?

The application of these criteria led to three kinds of packages:

- Software libraries.
- Datasets packaged with the software that produced them, and sometimes also with software that facilitates access to or analysis of the data.
- “Supplementary material” packages for a journal article, containing the top-level workflow up to the generation of all figures and tables.

We did not encounter any situation where it would have been useful to publish datasets without any accompanying software. For all datasets, there was some software that logically belonged to the data as an aid in its interpretation. However, this observation may be related to the fact that all the computational studies performed within the ActivePapers framework were of methodological nature, i.e. contained the development of new computational methods. As an example, reference 68 is a database of partial protein structures. They were constructed by downloading complete protein structures from the Protein Data Bank ^1^ and selecting parts that have specific structural features. The ActivePaper file includes the script that performs the download and the substructure extraction. This script is a detailed documentation of the selection criteria, and therefore valuable for anyone wishing to use the database.

One problem encountered in the course of these research projects is the relatively low size limit that today’s digital repositories impose on archived files. Zenodo ^5^ provides the most generous limit of 2 GB per file. However, the input data for one study ^71^ contains ten Molecular Dynamics (MD) trajectories for lysozyme in solvent, and requires 10 GB of storage even in compressed form. Since these data were not essential for the subsequent analysis step, which requires only the rigid-body motion of the protein, they were removed from the published files. The alternative would have been to publish each MD trajectory separately as an ActivePaper, and use DOI-based references in the analysis step to refer to this data. The main drawback to this approach is the additional effort required for dividing the data into small packages and publishing them separately.

ActivePapers proposes another mechanism to reduce file sizes: the deletion of recomputable datasets. Any dataset that was generated by a program stored in an ActivePaper can be replaced by a dummy dataset that retains only the dependency metadata. The full dataset can be recomputed on demand, or automatically when another program tries to read it. Recomputation consists in re-running the program that generated the dataset initially. This mechanism makes sense only if the replicability of a dataset is guaranteed. In practice, this applies to any program that does not use floating-point operations and whose input data is read exclusively from the ActivePapers universe. The ActivePapers implementation tests the second condition but cannot verify the first.

### Future developments

The existence of two distinct ActivePapers platforms is a historical accident that is clearly not desirable. The envisaged solution is a split of the ActivePapers platform definition into two parts: a data publishing system, which defines the HDF5 conventions for ActivePapers, in particular the metadata, and a code execution system that defines how specific datasets in ActivePapers are interpreted as executable code. Only the second part would differ between the current two implementations, and its separation also opens the way for additional execution systems for other code representations.

One line of future development is an integration of a narrative into the computational methods stored in an ActivePaper. Work on integrating the ActivePapers Python edition with the Jupyter project ^30^ (formerly the IPython notebook) is underway. Unfortunately, non-fundamental technical issues make this a non-trivial project: the various components (HDF5, Python, Jupyter) have different and conflicting requirements and restrictions concerning concurrency. Aside from these software engineering issues, the main question to be solved is how to reconcile the interactivity of the notebook approach with the permanence requirements of the scientific record. The coherence of code and results in a notebook is guaranteed only if it has been executed linearly from start to end. Any interactive manipulation results in general in a non-replicable state. Two solutions are currently explored. The first solution marks notebooks as non-replicable except when they have been executed linearly with no subsequent modification. No ActivePaper containing such non-replicable notebooks should be accepted by a digital repository. The second solution is to record all interactive code execution in a log, which is stored in the ActivePaper and can be re-executed later. After a complete linear execution of the notebook code, the log of interactive executions can be deleted.

Another direction for future developments explores how to provide a realistic transition from today’s scientific computing environments to future ones that take into account the needs for publishing and archiving computations. One important advantage of the ActivePapers approach in this context is that the minimal requirements for adopting it are modest: any software tool that can work with the ActivePapers file format, which is HDF5 plus a small set of conventions, can read and write publishable datasets. With a very small additional effort, software tools can be adapted to handle ActivePapers metadata and thus ensure dependency and provenance tracking. None of this requires that the software live inside the ActivePapers platform. The challenge for future ActivePapers developments is to facilitate the transition of computational models and methods from subroutines hidden inside software tools to specifications that become part of the scientific record.

## Conclusion

Experience with the two current implementations of the ActivePapers idea has shown that all of the requirements defined at the outset can be fulfilled and that the approach of publishing data and code packages works in practice. In particular, the ActivePapers project has shown that installation-free software deployment and long-time software preservation are possible, contrary to a common belief in the scientific computing community. ActivePapers can achieve these goals because the scientific contents of software take the form of pure computations, which are possible identically in all computational environments. The technological basis is a precise and stable definition for a computational platform, which includes a representation for executable code and a runtime library for interaction with the computational environment.

Experience with ActivePapers in real research projects has led to further important observations:

- Once software has a well-defined representation, it becomes a piece of data that can and should be handled exactly like any other scientific dataset.
- Packaging data and software together is natural in many situations because of the close relation between them.
- Installation-free deployment turns replication into a mechanical procedure that can be automated.
- Reuse of published data, including software, is the computational equivalent of a citation. It can be implemented using technology developed for citation (DOIs), and thus easily integrated into science metrics.

However, the ActivePapers experience has also shown that the majority of today’s scientific software does not fulfill the conditions for inclusion in the ActivePapers framework, nor in any similar framework based on the same design goals. There is in fact a significant gap between the requirements for the preservation and installation-free deployment of software and the dominant software development technologies in use today. The only existing computational platform that is clearly sufficient for implementing the ActivePapers approach is the JVM, which is not very popular in computational science, partly for real technical deficiencies, but partly also due to false perceptions of its performance. The CLI platform, which is currently evolving towards improved portability, is a second candidate. However, both of these platforms are based on a security model that requires automatic memory management. This is incompatible with the basic premises of the C and C++ languages, in which much of today’s scientific software is written. While compilation of C to JVM bytecode is possible (see e.g. 75), porting real C code represents an important effort and the results are often not satisfactory in terms of performance. Unless new platform candidates such as Google’s PNaCl turn out to be workable solutions, it seems like the scientific community will have to make a choice between the continued use of legacy software and the transition to a software development infrastructure that supports the replicability, reproducibility, and preservation of computational research.

Independently of the choice of a suitable computational platform, the separation of computational models and methods from software tools mentioned above is an important step towards reproducibility (through improved human understanding) and preservation of computer-aided research. Computational tools must in fact evolve with the progress of technology in order to remain useful to the communities that develop and apply them ^76^. The documentation and preservation of models and methods is much easier if they can evolve on the slower timescale of scientific progress. Such a separation is also completely analogous to how other digital content is handled. We archive articles in PDF/A format, movies in MPEG3 format, or protein structures in mmCIF format because these formats are well documented and allow anyone, at any point in time, to interpret the archived contents, even if today’s software tools are no longer usable because of the inherent instability of computational environments.
