# Supplementary material for: ActivePapers: a platform for publishing and archiving computer-aided research
Source: F1000Res. 2015 Jul 14;3:289. Originally published 2014 Nov 24. [Version 3] doi: 10.12688/f1000research.5773.3 (PMC4448745; doi:10.12688/f1000research.5773.3)
Supplement: Peer review history

# Peer review: Referee response for version 3

**DOI:** 10.5256/f1000research.7297.r15014

**Published:** 2016-07-14

**Authors:** Neil Chue Hong

**Affiliations:** 1 Software Sustainability Institute, University of Edinburgh, Edinburgh, UK

Thank you for the changes you have made in the two subsequent revisions to this article. I believe they have addressed my major comments from my review of the first version of this paper, and I therefore now approve this paper.

 Thank you as well for your detailed responses to reviewers comments.

I have read this submission. I believe that I have an appropriate level of expertise to confirm that it is of an acceptable scientific standard.

# Peer review: Referee response for version 3

**DOI:** 10.5256/f1000research.7297.r9500

**Published:** 2015-07-15

**Authors:** Carl Boettiger

**Affiliations:** 1 Center for Stock Assessment Research, Department of Applied Mathematics and Statistics, University of California, Santa Cruz, Santa Cruz, CA, USA

The author's comments and revisions appropriately address the issues I have raised.  I'm happy to recommend this for approval.

I have read this submission. I believe that I have an appropriate level of expertise to confirm that it is of an acceptable scientific standard.

# Peer review: Referee response for version 2

**DOI:** 10.5256/f1000research.6638.r8525

**Published:** 2015-05-28

**Authors:** Carl Boettiger

**Affiliations:** 1 Center for Stock Assessment Research, Department of Applied Mathematics and Statistics, University of California, Santa Cruz, Santa Cruz, CA, USA

The paper by Hinsen seeks to describe the ActivePapers project. The paper is clearly written and technically sound, and I am happy to recommend it for indexation. None the less I think the paper could be improved considerably in two areas. The first would be to improve the focus of the paper on the ActivePapers approach and motivation and less on much broader issues.The ActivePapers approach does many things in common with similar approaches, and some things differently. Having such case studies that identify both areas of consensus and highlight disagreement in approaches to reproducibility are very valuable, but this paper does not identify which is which. Second and closely related is a more thorough acknowledgement of related technology with more direct comparison of the similarities and differences. I explain both of these concerns in more detail below.  

The primary weakness of this paper is one of scope or focus. While I appreciate the importance of placing the paper in a broad context, the paper should spend somewhat less time discussing very generic issues more appropriate to a review (though given the rapidly changing landscape I am not sure that an up-to-date review of current technology and thinking in this area exists). More importantly, the paper should do much more to identify the particular challenges that the ActivePapers approach seeks to address that are either not addressed or approached very differently by other work in this area.This is most obvious in the conclusions.  The Introduction summarises the main contribution in six bullet points, the Conclusion does so with four somewhat different bullet points. Some of these are statements in which there is broad consensus in the reproducible research community or even the scientific community more broadly; others represent areas of important divisions. Similarly, some of these conclusions highlight challenges that are tackled very directly by the ActivePapers technology while not being addressed as widely elsewhere, while others highlight issues addressed by a wide array of existing approaches. As the author is reporting on a case study and not a broader survey or meta-analysis, some of the more sweeping generalizations seem out of place here (however much I also agree with them!) I suggest the paper would be stronger if the author more clearly outlined what conclusions come from the ActivePapers implementation directly, along with the evidence that supports those conclusions, and what conclusions represent opinion or position statements.  

The emphasis of the ActivePapers approach appears to be on the value provided by bytecode platforms such as the JVM for scientific computational *reproducibility*; while also recognizing that the JVM approach does not provide the necessary and sufficient tools for scientific computational *research*, which typically uses software not available for a JVM. For instance, this approach provides portability across platforms and bytecode written for a JVM has remained executable for decades, while compromising on details such as architecture-specific nature of performing floating-point computations. The author seeks to address this in part through an alternative Python implementation, but laments that external library dependencies required to do research in Python and the difficulties in tracking strong versioning available in the bytecode implementation. While I appreciate the author raising this often-overlooked issue of external library dependencies, which impacts Python, R, and many commonly used research languages, I worry that the paper may be overstating the concern. For one, the author does not present any evidence as to how often changes in these system dependencies really impact the reproducibilty of the code. More to the point, this is a problem which is 'turtles all the way down': just as capturing only the python layer misses possible changes in the system dependencies, even the JVM abstraction doesn't capture differences in very low-level elements or machine hardware. These differences are no doubt irrelevant to most researchers, (though perhaps not those studying the performance of algorithms on different architectures) but then from the perspective of most researchers the system level libraries are equally irrelevant. I suggest the author clarify when the focus on bytecode is essential and when it is less likely to be important than just capturing the layers that are more dynamic and closer to the researcher, as in both the python implementation and in many other similar efforts.   

The ideal requirements of an ActivePaper are neither precisely defined nor adequately motivated. Depending on the interpretation of these, I might name many examples of research implementations that meet these objectives or none at all.  Similarly, there are many other key features that are captured by the implementation of the ActivePapers project (and other efforts) that are not enumerated here. Taking them in turn:

1. "... should contain a combination of data, code, and narrative"
  What does "contain" mean?  Is a link to external data sufficient?  Elsewhere we learn that ActivePapers can import data from the network -- a very sensible thing as research frequently depends on previously published data and best-practices emphasize the importance of accessing the canonical, raw data. When should "contain" mean only a link, and when should it mean a bitwise representation in the HDF5 object?
2. "... always produce exactly the same result at the bit level"
  Much work in this area has not focused on the bit level, and it is unclear to me if the bit level is really the ideal criterion.  As the author notes, this is not met by the Python implementation of ActivePapers, which nonetheless may have many advantages in meeting the needs of more users.
3. "Any code stored in an ActivePaper should be safe to execute"
  The paper does well to raise security concerns, but these are largely orthogonal to the other issues discussed here.  However, the discussion is far too limited to illustrate what security concerns are and are not addressed.  Moreover it is not clear that such solutions need to be part of the platform that provides these other objectives, rather than being managed within existing security best-practices for a particular context. (i.e. if the emphasis is on isolation from the rest of the computing environment, containers, jails, virtualization, or a host of other options can be used; moreover it is not clear that such isolation is always necessary in this context any more than in the rest of the computing environment).
4. "Contain metadata for provenance & reproducibility"
  This is quite vague.  What metadata is and is not captured by the ActivePapers implementation is never clearly specified.  Moreover, there is no mention of providing this metadata in any of the several existing standards that would facilitate its reuse.  

These objectives would be made both more precise and more interesting if discussed in the context of similar efforts to provide technology for reproducible computation.  For instance, the practice of combining data, code, and narrative in context of scientific papers goes back at least to Gentleman and Temple Lang (2004), where the Sweave/R package approach has been frequently applied in published research. The discussion of virtualization makes no mention of the role of DevOps approach in addressing many of these issues, as described by Clark *et al. *(2014) or by more recent, lightweight alternatives to virualization such as containerization (as implemented by Docker but also by others in the scientific context, see the approach taken at CERN: http://arxiv.org/abs/1407.3063). Understandably this paper need not review all other technology, but where it does so it would be useful to map these more clearly to the four criteria above. What criteria are being met by the other approaches the author has considered? What are being only partially met?  What are missing? The current section "Tools for reproducible research" is too cursory an overview and is not tied back to the criteria or strengths/weaknesses of the ActivePapers approach.

I have read this submission. I believe that I have an appropriate level of expertise to confirm that it is of an acceptable scientific standard, however I have significant reservations, as outlined above.

# Response

**Published:** 2015-06-05

**Authors:** Konrad Hinsen

**Affiliations:** Centre de Biophysique Moléculaire (CNRS), France

There are many valid and useful remarks in this review, which I will take into account in a revision.

The one issue where I don't expect to be able to improve much is the scope and focus of the paper, which I have already tried to improve in the second version. The fundamental problem is that the field of reproducible research methodology is young, changing rapidly, and pursued independently in several domains of computational research which in addition have differing requirements. I am not aware of any review I could refer to for background information, and unlike the referee I am not sure that one can safely speak of any concensus beyond the general principle that reproducibility is important.

I might consider splitting the paper into two, one review and one dedicated to the ActivePapers project. However, to be really useful, a review would have to be co-authored by researchers from different background.

Concerning the issue of the stability of external library dependencies, a study of the importance of this problem would indeed be interesting, but again this would have to be done in collaboration by researchers from diverse background to be representative. The most telling personal anecdotical evidence that I can provide is the fact that a single change in NumPy 1.9 broke all the research code that I have published between 1997 and 2012. I have documented this issue in a blog post.

As for the "turtles all the way down", this is exactly what needs to be fixed in order to solve the software preservation and reproducibility problem. There needs to be a stable layer somewhere in the tower of code representations. The JVM designers tried, succeeded, and then changed their minds for obtaining a short-term market share benefit. In my opinion, this shows that the problem can be solved, but only if it is treated as a priority in the computational science community.

I do not agree that this is a matter of relevance to specific user communities. I suspect most scientists consider the problem irrelevant until it touches their own code. In the absence of a stable computational platform at a sufficiently low layer to cover all scientific code, nobody is safe from such problems. What happened to me with NumPy can happen to anyone who relies on code that is maintained by someone else.

As for the ideal requirements of an ActivePaper, I will try to improve on the motivations, but I doubt that the requirements can be precisely defined at this time. They evolved significantly since the beginning of the project. This is probably another reason for the lack of focus in some parts of the paper: a narrative in historical order is desirable to explain the motivations behind each choice, but messy because at different points in time the requirements were not the same.

As for the reviewer's point 1, "should contain" should actually read "should be allowed to contain". Both inclusion and linking are important to have as an option, and both are supported by the ActivePapers platform, but the choice between them remains with the author of each published work.

As for point 2, bit-level reproducibility is the only form of reproducibility that can be guaranteed at the level of a computational platform. A concrete computational study may not require that much, but any "close enough" criterion is necessarily application-specific. Interestingly, most of today's computational platforms do not make any promise about floating-point reproducibility, creating the same problem as with external libraries: the reproducibility of my code relies on something that is completely out of my control.

Point 3, security issues, and point 4, metadata, do indeed require a better treatment in the paper. In short, the JVM edition has good security support (inherited from the JVM libraries), whereas the Python edition has almost none, because the Python platform has none. Metadata handled by the ActivePapers platform will be documented in detail in the revision. Note that users can add any metadata they like, following any standards they consider useful. The metadata defined by the platform is limited to what can be automatically generated from the history of an ActivePaper.

# Peer review: Referee response for version 2

**DOI:** 10.5256/f1000research.6638.r7867

**Published:** 2015-03-18

**Authors:** Mercè Crosas, Vito D'Orazio

**Affiliations:** 1 Institute for Quantitative Social Sciences, Harvard University, Cambridge, MA, USA; 2 Harvard University, Cambridge, USA

This article has been thoroughly revised. Its purpose is clear, and its finding are supported by a mixture of literature and software review and experience with ActivePapers. We have two minor suggested revisions.

First, the four bullets in the conclusions could also be worked into the introduction. We see these as well-stated, important points to make early in the paper. For example, the first bullet---thinking of a software representation as a dataset and archiving it accordingly---is a simple, intuitive way to think about much of what is discussed in this paper.

Second, on page 4, the author says "many practitioners consider the idea of preserving scientific software for many years unrealistic, and some even argue that it is unnecessary because computational methods change so rapidly that their long-term preservation is of no interest." This statement needs citations.

We have read this submission. We believe that we have an appropriate level of expertise to confirm that it is of an acceptable scientific standard.

# Peer review: Referee response for version 1

**DOI:** 10.5256/f1000research.6172.r6988

**Published:** 2015-01-26

**Authors:** Mercè Crosas, Vito D'Orazio

**Affiliations:** 1 Institute for Quantitative Social Sciences, Harvard University, Cambridge, MA, USA; 2 Harvard University, Cambridge, USA

**Summary of the Paper**

This article summarizes arguments in support of reproducibility for scientific research, specifically with respect to computational science. It raises important issues about scholarly communication and reproducibility of previous research work, and it presents ActivePapers as a solution to many problems associated with reproducibility.

**Reaction**

Upon reading the article, our reaction is twofold.  First, we do not understand the author's primary purpose. Is this a review article or a research article? Is this an article summarizing arguments in support of reproducibility? Is it making the case for ActivePapers? Second, regardless of the primary purpose, we believe the work misses key existing technologies and practices.

**Structural Issues**

Is this a review article or a research article?  If this is a review article, replicability and reproducibility should be discussed in more detail, and ActivePapers in less. If this is a research article, we suggest less review of the ActivePapers platform, and more on its contribution and the ways it addresses problems that other systems do not.

The introduction states, *"the work presented in this article takes the opposite approach of starting from the requirements of the scientific record and exploring how software and electronic datasets need to be prepared in order to become useful parts of this record."* What are the requirements of the scientific record? They seem scattered throughout the next few sections, such as " *the most basic requirement for building confidence in computational results is total transparency"* (p. 3). The philosophy of science literature might be helpful in summarizing the basic requirements.

Based on our reading, we do not think the main contributions of this paper are the bulleted items in the introduction, but rather the ideal platform requirements on page six. These logically flow from a discussion of the requirements of the scientific record followed by a discussion of tools for reproducible and replicable research.

**On Data Reuse**

There are preservation practices and tools, which follow the approach widely used in Libraries, that help to make a dataset reusable in the longer term:

First, researchers are encouraged to use and share datasets in formats commonly used by their discipline, and when possible, formats that do not depend on proprietary software. When the data file format depends on a specific software (or is not considered a preservation format), there exist software tools that convert the proprietary format to a preservation format. For example, for tabular data in SPSS and STATA format which depend on a specific statistical package, the files can be converted to a plain text (tab-delimited or CSV file) plus a metadata file (in XML or JSON format) that contains information about the columns in the original tabular data file. Information is not lost and it can be re-combine to generate an SPSS or STATA file.

This preservation feature can be found in public data repositories such as the Dataverse repository software ( dataverse.org). There are software projects that focus on other similar automated preservation tools to re-format data files into preservation formats and provide additional preservation metadata. One example is Archivematica.

These preservation tools are important for data repositories if they want to make their data accessible and reusable in the future, when the original software might be obsolete.

**On Methods/Code/Software**

One approach on sharing the code used to model/analyze a research work is by using an open-source language like R, where the models and packages are shared and disseminated through a common R package repository (CRAN). Some R packages have explored solutions for reproducibility by tracking the detailed information on what model/code ran and the computing configuration (see http://arxiv.org/pdf/1501.02284.pdf).

Also, there are on-going efforts in reproducibility that support hosting code and provide executable functionality to run in the cloud ( http://researchcompendia.org/).

**Minor Issues**

The word *conservation* should be replaced with *preservation*. Conservation generally implies the careful maintenance of a finite resource, while preservation implies the protection of a thing in order to keep that thing as is.

We recommend reviewing the work and guidelines provided by the Data Citation Principles: https://www.force11.org/datacitation which can apply to software as well, and should help towards giving due importance to software and providing formal long term access and reuse, if the principles are followed.

In the conclusion: *"the main reason is that most of the computing technology used by scientists was developed outside of scientific research, for domains of application where replicability is not important."* Is this statement supported?

**Indexability**

This article requires considerable work to be indexed. First, clarify the type of article that is being written, and restructure accordingly. Second, the author misses some key existing technologies and practices that should be discussed.

We have read this submission. We believe that we have an appropriate level of expertise to confirm that it is of an acceptable scientific standard, however we have significant reservations, as outlined above.

# Response

**Published:** 2015-02-24

**Authors:** Konrad Hinsen

**Affiliations:** Centre de Biophysique Moléculaire (CNRS), France

The article has been completely restructured in order to make its primary goal stand out: report the lessons learned from developing and using a platform for publishing and archiving computational science. The revised article starts with an analysis of the needs of the scientific record, deduces technical requirements for a platform designed to meet these needs, and then describes the concrete technical choices made in the two implementations of ActivePapers, followed by a report of the lessons learned from its use. The review of existing technology and the description of the ActivePapers platform are still quite long, but inevitable because I am not aware of any other work I could refer the reader to for this essential background information. Even for fundamental issues such as the requirements of the scientific record, I did not find any publication discussing the specific problems of software and datasets. Software in science is almost always discussed exclusively from the point of view of its utility, ignoring its role as an encoding of scientific knowledge.

I fully agree with the reviewers' point of view on data reuse and how to improve it, but I don't see much adoption of these techniques in my scientific environment. Scientists prefer the simplicity of just publishing the files they have on their Web site or on a no-questions-asked digital repository. It's encouraging to see that other domains have succeeded in establishing better habits.

I was not aware of the work by Becker et al. on the tools switchr and GRANbase, which are based on very similar ideas as ActivePapers. A short comparison has been added to the revised article. I was aware of Research Compendia, which however does not address software preservation or even execution in its current version. I do mention the two cloud-hosting sites with on-line execution that I know of: Exec&Share ( http://www.execandshare.org) and Elsevier's Collage system ( http://collage.elsevier.com).

The term "conservation" has been replaced by "preservation" in the revision, and the related issues of software preservation and software deployment are now the common thread around which the presentation is organized. Existing work on the preservation of electronic artifacts has been integrated into the discussion.

# Peer review: Referee response for version 1

**DOI:** 10.5256/f1000research.6172.r6774

**Published:** 2014-12-09

**Authors:** Neil Chue Hong

**Affiliations:** 1 Software Sustainability Institute, University of Edinburgh, Edinburgh, UK

I, Neil Chue Hong, have reviewed this research article following the principles set out in the Open Science Peer Review Oath v1 (DOI: 10.12688/f1000research.5686.1). 

This article by Konrad Hinsen discusses the very important issue of how we capture the detail of the dependencies and environment surrounding the software tools that we use, such that we can guarantee that the tools may be used in the future to replicate research and reuse the tools. Overall, it is a comprehensive summary of most of the area.

However as it stands, I believe that the article could be significantly improved by deciding whether the paper is to be a fully comprehensive "state of the art" summary, looking at the specific advantages and disadvantages of each approach; or one which sets out a shorter summary of the state of the art (as is present in the article in this version), and then goes on to describe the lessons learned from the ActivePapers work, in which case I would suggest a change in title to reflect the emphasis on ActivePapers as a primary example of a platform for publishing and archiving computer aided research. Therefore I am marking this as "Approved with reservations" as it requires a structural change, rather than because I believe the work contained in it is not technically sound.

In both scenarios for how the article could be rewritten, I believe the article would benefit from the use of more devices to highlight key points and comparisons, for instance by use of tables to compare the effect of different types of platforms on the ability to define environmental dependencies, or linkage with input data.

There is one important area of research that should be covered in the discussion of the state of the art - that around  "significant properties" of software. In particular, the work of Brian Matthews at STFC in this area has previously considered the issue of capturing and prioritising details of both the environment, and how a computational tool is expected to function, thus forming a theoretical basis for describing the way that many current implementations ranging from dependency managers like Maven, through configuration management tools like Docker, Vagrant and Conda, to virtualisation. See:

1. http://www.jisc.ac.uk/media/documents/programmes/preservation/spsoftware_report_redacted.pdf
2. http://ijdc.net/index.php/ijdc/article/view/148

In terms of other areas where I felt that additional discussion would have provoked more debate, these would be around the trade-offs surround floating point operations, a discussion of other bytecode platforms, and around the long lifetime of successful pieces of software, in particular around trust and how it is mechanically/technically checked.

This last point is illustrated in this example from random sampling: https://cryptogenomicon.wordpress.com/2014/10/13/cryptic-genetic-variation-in-software-hunting-a-buffered-41-year-old-bug/

As minor points that I believe would improve the papers I would suggest the following:

- The term "Web repositories" for platforms like FigShare and Zenodo is not commonly used - indeed, it is more commonly used to refer to repositories of web pages. I would suggest the more commonly used "digital repositories" term, or perhaps "web accessible third party digital repositories"?
- On page 3, it would be useful to describe what makes "Web repositories" better. I would suggest it is cost (most are free for openly licensed deposits) and the ability to generate citable DOI s
- On page 3, when talking about the versioning used by *F1000Research*, it should be clarified that "a DOI refers permanently to a specific version of an article"
- On page 3, the author's example of the DSSP method still being widely used today could be seen to contradict the earlier arguments surrounding the inability of code to preserved effectively.
- Whilst I agree that as stated on Page 5 "This lack of a precisely defined and stable platform for executable code is also the root cause of non-replicability in computer-aided research" I feel that the author could discuss the tradeoffs (mostly optimisation and performance based) in more detail and put across their opinion of which are most important.
- On page 6, I had a little difficulty with the statement that "the scientific aspects of software are always pure computation". I think that I understand what the author means, but as written it makes me want to identify a counter example. In fairness, I haven't been able to find one yet.
- On page 7, I note that all the examples of ActivePapers I could find on FigShare have the author as a co-author on them. This means it is unclear whether ActivePapers Python edition is indeed suitable for the wide variety of scientific research areas, as it is unclear whether they represent a representative selection of the use cases for ActivePapers.
- I believe that the author could do more to support their statement that the fundamental distinction is between "computational tool" and "scientific content". This could probably be done by making it clearer how current platforms do or don't support this conceptual split, and whether those that do support the split lead to a more accurate ability to replicate research at a later date.

As a final note, I would say that it is the tacit convention in *all* science that published results are assumed correct unless there is clear evidence to suggest otherwise.

I believe that with some structural changes to give it a clear narrative emphasis, and better figures to present the information that this research article would provide significant information to the community in this area.

I have read this submission. I believe that I have an appropriate level of expertise to confirm that it is of an acceptable scientific standard, however I have significant reservations, as outlined above.

# Response

**Published:** 2015-02-24

**Authors:** Konrad Hinsen

**Affiliations:** Centre de Biophysique Moléculaire (CNRS), France

My goal was not to write a review article on the topic of computational reproducibility, but to present the conclusions from developing  ActivePapers and applying it in real-life research projects. The article has been restructured accordingly. The review of prior work has been shortened and reframed in the context of the ActivePapers project. The "lessons learned" from ActivePapers are presented in more detail.

I was not aware of the work of Brian Matthews on software preservation in the context of scientific computing. This is indeed very relevant and is mentioned in the revision. I have also expanded the discussion of bytecode platforms and floating-point operations. The discussion of bytecode platform also addresses the issue of trust and security.

The minor points have all been addressed in the revised manuscript.

Concerning the example of DSSP, it is the method but not the original code that is still widely used. Few users of today's code know that it differs from the originally published method. I do not know if the original code actually implemented the method described in the paper. It is no longer available and I have never seen it.

I have expanded my statement that "the scientific aspects of software are always pure computation" into a more detailed paragraph, and referred to it in several places in the article. I believe that this point is very important but often overlooked. The structure of an ActivePaper, combining software and the data it works one, makes it clear that only pure computations can be replicable. I then found out that this is common knowledge in other fields, such as programming language theory.

I have also expanded the discussion of the factors that have until now prevented the establishment of a stable platform for scientific computing. While performance is often quoted as an important factor, as the reviewer remarks, I do not think this can be backed up by much evidence. To the best of my knowledge, no attempt has been made to design and implement a high-performance yet rigorously defined platform, so it cannot be claimed that this is impossible. The problem is rather that for economical reasons, progress in computing happens as a sequence of small, localized changes: a revision of a language, then a new processor generation, a library update, etc. Each local change must work correctly and efficiently with the existing ecosystem of computing technology. A stable platform definition requires coordinated changes in several technological layers, which is difficult to achieve.

It is well possible that the only currently published ActivePapers are those that I cite, and for which I am a co-author. I know about a few other groups experimenting with ActivePapers, but they have not published any results in this form yet. However, I do not make the claim that ActivePapers is in its current form a good solution for all branches of computational science. Like any other tool, it was written with specific techniques and workflows in mind. Like for any other tool, only long-time experience will show how universallly applicable it is. Moreover, the difficulty of integrating legacy software is a real problem for adoption. This article doesn't pretend to do more that present the lessons learned from the applications that are cited.

I cannot support my opinion about the distinction between tools and scientific content by any empirical evidence, because to the best of my knowledge no existing framework or tool chain for scientific computing supports the implementation of such an approach.

I agree that the tacit assumption of correctness of published results is applied everywhere in science. The particularity of computational science is that this is usually impossible to verify, both for the authors of a scientific study and for their readers.
